# Supplementary material for: Splicing variation of BMP2K balances abundance of COPII assemblies and autophagic degradation in erythroid cells
Source: eLife. 2020 Aug 14;9:e58504. doi: 10.7554/eLife.58504 (PMC7473771; doi:10.7554/eLife.58504)
Supplement: Supplementary file 1. — Tables 1 and 2. List of proteins detected in BioID as proximal to BMP2K-L (1) or BMP2K-S (2) tagged with a mutant BirA biotin ligase (BirA*). Corresponding Gene symbols and Uniprot identifiers are provided. The list is ranked according to mean score between subtracted scores from N-terminally (N-tag) and C-terminally (C-tag) tagged baits. Table 3. List of shRNAs designed to deplete both (shBMP2K), or specific (BMP2K-L or BMP2K-S) BMP2K variants. Target nucleotide sequences as well as their locations on mRNA are provided. CDS – coding sequence, UTR – untranslated region. Table 4. List of gRNAs, non-targeting (gCtrl#1 and 2) or targeting BMP2K gene by CRISPR/Cas9 system (gBMP2K#1 and 2). When applicable, chromosomal position of base after cut by Cas9 as well as targeted DNA strand and location on gene are indicated. Table 5. List of primers used for assessing the levels of indicated human or mouse transcripts using qRT-PCR. Nucleotide sequences of both, forward and reverse primers are provided. [file elife-58504-supp1.docx]

**Cendrowski et al. SUPPLEMENTARY TABLES**

**Table 1. The ranked list of proteins detected in BioID using BMP2K-L as bait.**

| **Rank** | **Protein name** | **Gene symbol** | **Uniprot ID** | **Subtracted score C-tag** | **Subtracted score N-tag** | **Mean score** |
| --- | --- | --- | --- | --- | --- | --- |
| N/A | BMP-2-inducible protein kinase | BMP2K | Q9NSY1 | 14142.3 | 10216.0 | 12179.1 |
| 1 | E3 SUMO-protein ligase RanBP2 | RANBP2 | P49792 | 1908.5 | 1004.5 | 1456.5 |
| 2 | Histone H1.4 | HIST1H1E | P10412 | 682.8 | 807.0 | 744.9 |
| 3 | Epidermal growth factor receptor substrate 15-like 1 | EPS15R | Q9UBC2 | 573.8 | 422.5 | 498.1 |
| 4 | Histone H1.2 | HIST1H1C | P16403 | 199.0 | 740.5 | 469.8 |
| 5 | 40S ribosomal protein S30 | FAU | P62861 | 411.5 | 260.8 | 336.1 |
| 6 | MKL/myocardin-like protein 2 | MKL2 | Q9ULH7 | 304.8 | 265.3 | 285.0 |
| 7 | Guanine nucleotide-binding protein-like 3 | GNL3 | Q9BVP2 | 364.0 | 176.3 | 270.1 |
| 8 | 60S ribosomal protein L6 | RPL6 | Q02878 | 438.0 | 77.0 | 257.5 |
| 9 | RalBP1-associated Eps domain-containing protein 1 | REPS1 | Q96D71 | 115.5 | 307.3 | 211.4 |
| 10 | Transcription termination factor 1 | TTF1 | Q15361 | 178.0 | 209.0 | 193.5 |
| 11 | RANBP2-like and GRIP domain-containing protein 1 | RGPD1 | P0DJD0 | 206.8 | 177.8 | 192.3 |
| 12 | 40S ribosomal protein S8 | RPS8 | P62241 | 311.8 | 60.5 | 186.1 |
| 13 | Nucleolar protein of 40 kDa | ZCCHC17 | Q9NP64 | 252.3 | 50.5 | 151.4 |
| 14 | Arf-GAP domain and FG repeat-containing protein 1 | AGFG1 | P52594 | 145.3 | 147.3 | 146.3 |
| 15 | 60S ribosomal protein L26 | RPL26 | P61254 | 199.0 | 88.3 | 143.6 |
| 16 | Protein numb homolog | NUMB | P49757 | 146.5 | 127.0 | 136.8 |
| 17 | pre-rRNA processing protein FTSJ3 | FTSJ3 | Q8IY81 | 187.0 | 70.8 | 128.9 |
| 18 | Phosphatidylinositol-binding clathrin assembly protein | PICALM | Q13492 | 63.0 | 128.8 | 95.9 |
| 19 | 60S ribosomal protein L32 | RPL32 | P62910 | 117.8 | 67.8 | 92.8 |

**Table 2. The ranked list of proteins detected in BioID using BMP2K-S as bait.**

| **Rank** | **Protein name** | **Gene symbol** | **Uniprot ID** | **Subtracted score C-tag** | **Subtracted score N-tag** | **Mean score** |
| --- | --- | --- | --- | --- | --- | --- |
| N/A | BMP-2-inducible protein kinase | BMP2K | Q9NSY1 | 24769.3 | 6273.3 | 15521.3 |
| 1 | E3 SUMO-protein ligase RanBP2 | RANBP2 | P49792 | 7906.3 | 3021.3 | 5463.8 |
| 2 | Protein PRRC2C | PRRC2C | Q9Y520 | 2433.0 | 2461.5 | 2447.3 |
| 3 | Protein transport protein Sec16A | SEC16A | O15027 | 3005.8 | 1667.5 | 2336.6 |
| 4 | Ubiquitin-associated protein 2-like | UBAP2L | Q14157 | 2202.3 | 1675.5 | 1938.9 |
| 5 | Ataxin-2-like protein | ATXN2L | Q8WWM7 | 1231.8 | 974.5 | 1103.1 |
| 6 | Protein PRRC2A | PRRC2A | P48634 | 1187.3 | 901.8 | 1044.5 |
| 7 | Ankyrin repeat and KH domain-containing protein 1 | ANKHD1 | Q8IWZ3 | 1087.8 | 955.8 | 1021.8 |
| 8 | Double-strand break repair protein MRE11A | MRE11A | P49959 | 1739.3 | 51.5 | 895.4 |
| 9 | Protein PRRC2B | PRRC2B | Q5JSZ5 | 1092.5 | 573.5 | 833.0 |
| 10 | Nuclear fragile X mental retardation-interacting protein 2 | NUFIP2 | Q7Z417 | 443.0 | 1174.8 | 808.9 |
| 11 | RANBP2-like and GRIP domain-containing protein 1 | RGPD1 | P0DJD0 | 1063.5 | 548.8 | 806.1 |
| 12 | Ankyrin repeat domain-containing protein 17 | ANKRD17 | O75179 | 1245.3 | 351.3 | 798.3 |
| 13 | Epidermal growth factor receptor substrate 15-like 1 | EPS15R | Q9UBC2 | 1070.5 | 342.0 | 706.3 |
| 14 | Nuclear pore complex protein Nup214 | NUP214 | P35658 | 951.5 | 333.3 | 642.4 |
| 15 | RANBP2-like and GRIP domain-containing protein 8 | RGPD8 | O14715 | 960.5 | 277.8 | 619.1 |
| 16 | Phosphatidylinositol-binding clathrin assembly protein | PICALM | Q13492 | 551.5 | 668.5 | 610.0 |
| 17 | Eukaryotic translation initiation factor 4 gamma 1 | EIF4G1 | Q04637 | 807.8 | 406.3 | 607.0 |
| 18 | Ubiquitin-associated protein 2 | UBAP2 | Q5T6F2 | 823.8 | 356.0 | 589.9 |
| 19 | Clathrin interactor 1 | CLINT1 | Q14677 | 735.5 | 365.8 | 550.6 |
| 20 | Histone H1.4 | HIST1H1E | P10412 | 764.8 | 258.8 | 511.8 |
| 21 | Probable JmjC domain-containing histone demethylation protein 2C | JMJD1C | Q15652 | 615.8 | 298.3 | 457.0 |
| 22 | Tight junction protein ZO-1 | TJP1 | Q07157 | 748.5 | 147.3 | 447.9 |
| 23 | PERQ amino acid-rich with GYF domain-containing protein 2 | GIGYF2 | Q6Y7W6 | 590.3 | 303.3 | 446.8 |
| 24 | Trinucleotide repeat-containing gene 6B protein | TNRC6B | Q9UPQ9 | 356.3 | 501.5 | 428.9 |
| 25 | MKL/myocardin-like protein 2 | MKL2 | Q9ULH7 | 757.8 | 95.8 | 426.8 |
| 26 | 5'-3' exoribonuclease 1 | XRN1 | Q8IZH2 | 540.8 | 248.3 | 394.5 |
| 27 | Pericentriolar material 1 protein | PCM1 | Q15154 | 383.0 | 359.8 | 371.4 |
| 28 | Arf-GAP domain and FG repeat-containing protein 1 | AGFG1 | P52594 | 550.5 | 191.3 | 370.9 |
| 29 | Polyadenylate-binding protein 1 | PABPC1 | P11940 | 652.0 | 87.5 | 369.8 |
| 30 | Propionyl-CoA carboxylase alpha chain, mitochondrial | PCCA | P05165 | 432.5 | 289.8 | 361.1 |
| 31 | Eukaryotic translation initiation factor 4 gamma 3 | EIF4G3 | O43432 | 392.5 | 311.8 | 352.1 |
| 32 | Eukaryotic translation initiation factor 4E transporter | EIF4ENIF1 | Q9NRA8 | 291.8 | 345.5 | 318.6 |
| 33 | Segment polarity protein dishevelled homolog DVL-2 | DVL2 | O14641 | 491.3 | 78.5 | 284.9 |
| 34 | Segment polarity protein dishevelled homolog DVL-3 | DVL3 | Q92997 | 477.0 | 86.5 | 281.8 |
| 35 | Cold shock domain-containing protein E1 | CSDE1 | O75534 | 400.3 | 158.3 | 279.3 |
| 36 | Protein LAP2 | ERBB2IP | Q96RT1 | 287.3 | 257.5 | 272.4 |
| 37 | Alstrom syndrome protein 1 | ALMS1 | Q8TCU4 | 253.0 | 259.5 | 256.3 |
| 38 | Trinucleotide repeat-containing gene 6A protein | TNRC6A | Q8NDV7 | 148.0 | 352.0 | 250.0 |
| 39 | Pumilio homolog 1 | PUM1 | Q14671 | 172.5 | 305.8 | 239.1 |
| 40 | YTH domain-containing family protein 3 | YTHDF3 | Q7Z739 | 261.8 | 173.8 | 217.8 |
| 41 | CCR4-NOT transcription complex subunit 1 | CNOT1 | A5YKK6 | 290.3 | 137.8 | 214.0 |
| 42 | Guanine nucleotide-binding protein-like 3 | GNL3 | Q9BVP2 | 155.8 | 269.0 | 212.4 |
| 43 | Protein numb homolog | NUMB | P49757 | 214.0 | 205.3 | 209.6 |
| 44 | Probable helicase with zinc finger domain | HELZ | P42694 | 239.8 | 149.0 | 194.4 |
| 45 | Nucleolar protein of 40 kDa | ZCCHC17 | Q9NP64 | 273.0 | 102.3 | 187.6 |
| 46 | Ataxin-2 | ATXN2 | Q99700 | 193.0 | 172.0 | 182.5 |
| 47 | 60S ribosomal protein L36 | RPL36 | Q9Y3U8 | 300.5 | 52.3 | 176.4 |
| 48 | Protein SMG7 | SMG7 | Q92540 | 263.8 | 80.3 | 172.0 |
| 49 | YTH domain-containing family protein 1 | YTHDF1 | Q9BYJ9 | 62.3 | 252.5 | 157.4 |
| 50 | HAUS augmin-like complex subunit 6 | HAUS6 | Q7Z4H7 | 251.5 | 56.8 | 154.1 |
| 51 | Utrophin | UTRN | P46939 | 246.0 | 54.0 | 150.0 |
| 52 | Protein transport protein Sec24B | SEC24B | O95487 | 232.3 | 55.3 | 143.8 |
| 53 | ELKS/Rab6-interacting/CAST family member 1 | ERC1 | Q8IUD2 | 233.5 | 52.5 | 143.0 |
| 54 | YTH domain-containing family protein 2 | YTHDF2 | Q9Y5A9 | 130.5 | 150.5 | 140.5 |
| 55 | Nuclear pore complex protein Nup133 | NUP133 | Q8WUM0 | 195.8 | 82.5 | 139.1 |
| 56 | 60S ribosomal protein L35 | RPL35 | P42766 | 164.8 | 105.5 | 135.1 |
| 57 | Methylcrotonoyl-CoA carboxylase subunit alpha, mitochondrial | MCCC1 | Q96RQ3 | 53.8 | 209.5 | 131.6 |
| 58 | Roquin-1 | RC3H1 | Q5TC82 | 153.8 | 106.0 | 129.9 |
| 59 | R3H domain-containing protein 1 | R3HDM1 | Q15032 | 60.0 | 176.0 | 118.0 |
| 60 | Insulin-like growth factor 2 mRNA-binding protein 2 | IGF2BP2 | Q9Y6M1 | 115.3 | 120.0 | 117.6 |
| 61 | Far upstream element-binding protein 3 | FUBP3 | Q96I24 | 70.3 | 164.8 | 117.5 |
| 62 | Intersectin-1 | ITSN1 | Q15811 | 152.8 | 75.0 | 113.9 |
| 63 | BCL-6 corepressor | BCOR | Q6W2J9 | 80.0 | 117.0 | 98.5 |
| 64 | Pumilio homolog 2 | PUM2 | Q8TB72 | 97.5 | 88.3 | 92.9 |
| 65 | Transcription termination factor 1 | TTF1 | Q15361 | 116.8 | 55.3 | 86.0 |
| 66 | Roquin-2 | RC3H2 | Q9HBD1 | 71.0 | 98.8 | 84.9 |
| 67 | Epidermal growth factor receptor substrate 15 | EPS15 | P42566 | 56.5 | 112.5 | 84.5 |
| 68 | mRNA-decapping enzyme 1A | DCP1A | Q9NPI6 | 108.0 | 57.3 | 82.6 |
| 69 | Adenomatous polyposis coli protein | APC | P25054 | 80.8 | 81.8 | 81.3 |
| 70 | OTU domain-containing protein 4 | OTUD4 | Q01804 | 89.0 | 60.8 | 74.9 |
| 71 | WASH complex subunit FAM21C | FAM21C | Q9Y4E1 | 67.8 | 80.5 | 74.1 |
| 72 | ADP-ribosylation factor GTPase-activating protein 1 | ARFGAP1 | Q8N6T3 | 83.0 | 50.8 | 66.9 |
| 73 | Poly(rC)-binding protein 2 | PCBP2 | Q15366 | 72.5 | 53.0 | 62.8 |

**Table 3. shRNAs used for depletion of all or specific BMP2K splicing variants**

| **shRNA name** | **Target sequence** | **Targeted location on mRNA** |
| --- | --- | --- |
| shBMP2K | GACTGTGCTGTTAATTCAATT | exon 3 of BMP2K-L and BMP2K-S, CDS |
| shBMP2K-L | CATCCGTGCTGATCACAAT | exon 16 of BMP2K-L, CDS |
| shBMP2K-L#2 | GCACTGCAAACCCTATCAA | exon 16 of BMP2K-L, CDS |
| shBMP2K-S | GTAGCTATTAAACCCAATA | exon 14 of BMP2K-S, 3’ UTR |
| shBMP2K-S#2 | GCTTGATAGTAGCTATTAA | exon 14 of BMP2K-S, 3’ UTR |

**Table 4. gRNAs used for CRISPR/Cas9-mediated gene inactivation**

| **gRNA name** | **Target sequence** | **Chromosomal Position of Base After Cut** | **Strand** | **Targeted location on gene** |
| --- | --- | --- | --- | --- |
| gCtr#1 | ACGGGCGGCTATCGCTGACT | - | - | - |
| gCtr#2 | CGCTTCCGCGGCCCGTTCAA | - | - | - |
| gBMP2K#1 | GTAACGAGAATTGTCTGGGA | 78851015 (*chr4*) | antisense | exon 7 |
| gBMP2K#2 | GAAATGATCAACCTTTATGG | 78847233 (*chr4*) | sense | exon 6 |

**Table 5. Sequences of primers used for qRT-PCR**

| **Transcript name** | **Forward primer** | **Reverse primer** |
| --- | --- | --- |
| ANK1 (human) | TACTCGCTGTCACCCTC | GTACTGGTCTGCACGTAG |
| BMP2K-L (mouse) | CAGATAAGACTGTAGACCTGC | TCGCTGATCTTTACATAGTGG |
| BMP2K-S (mouse) | TAGTGGACTCTTCTTACGGT | AATAAGCCTTCAAGTGTCCC |
| GAPDH (human) | CATGTTCGTCATGGGTGTGAACCA | GTGATGGCATGGACTGTGGTCAT |
| GYPA (human) | AGTTACATCTCATCACAGACA | TTCCTCTTCTGGAGGGTAAA |
| HBA1 (human) | GGACCCGGTCAACTTCAA | TAACGGTATTTGGAGGTCAG |
| HBB (human) | GGAGAAGTCTGCCGTTAC | GATCCCCAAAGGACTCAAAG |
| HBE1 (human) | ACTTCCTTTGGAGATGCTATTA | AGAATAATCACCATCACGTTAC |
| p62/SQSTM1 (human) | GAATCAGCTTCTGGTCCATCGG | GCTTCTTTTCCCTCCGTGCT |
| RPL19 (mouse) | AGGCATATGGGCATAGGGAAGAG | TTGACCTTCAGGTACAGGCTGTG |
| SEC16A (human) | GACTCAGTCAGCCAGGA | AATGGGCCGTCAGGG |
| TFRC (human) | ACGTCGCTTATATTGGGATG | TCACGAGGGACATATGAATTT |
| TFRC (mouse) | TTAAATTCAGCAAAGTCTGGC | CTCCACTGGGTCTAAGTTAC |
| TFR2 (human) | ACTTCCTTTGGAGATGCTATTA | AGAATAATCACCATCACGTTAC |
